# Cytokine Profiles as Predictive Biomarkers of Disease Severity and Progression in Engineered Stone Silicosis: A Machine Learning Approach

**DOI:** 10.3390/diagnostics15182413

**Published:** 2025-09-22

**Authors:** Daniel Sanchez-Morillo, Ana Martín-Carrillo, Blanca Priego-Torres, Iris Sopo-Lambea, Gema Jiménez-Gómez, Antonio León-Jiménez, Antonio Campos-Caro

**Affiliations:** 1 Department of Automation Engineering, Electronics and Computer Architecture and Networks, School of Engineering, University of Cadiz, 11519 Cadiz, Spain; 2 Biomedical Research and Innovation Institute of Cadiz (INiBICA), 11009 Cadiz, Spain; 3 Research Unit, Puerta del Mar University Hospital, 11009 Cadiz, Spain; 4 Pulmonology Department, Puerta del Mar University Hospital, 11009 Cadiz, Spain; 5 Genetics Area, Biomedicine, Biotechnology and Public Health Department, School of Marine and Environmental Sciences, University of Cadiz, 11510 Cadiz, Spain

**Keywords:** engineered stone, silicosis, cytokines, biomarkers, machine learning

## Abstract

**Background/Objectives**: Silicosis caused by dust from engineered stone (ES) exposure is an emerging occupational lung disease that severely impacts respiratory health. This study aimed to analyze the association between cytokine profiles and disease severity and progression in patients with engineered stone silicosis (ESS) to assess their potential as biomarkers of progression and their usefulness to stratify risk. **Methods**: A longitudinal study was conducted with a seven-year follow-up (2017-2024) on 72 workers with simple silicosis (SS) or progressive massive fibrosis (PMF), all with a history of cutting, polishing, and finishing ES countertops. Data on lung function and levels of 27 cytokines were collected at four control points. Machine learning (ML) models were built to classify the disease stage and predict its progression. **Results**: 39% of patients with SS progressed to PMF. Significant differences in the expression of some cytokines were observed between ESS stages, suggesting a role in the evolution of the inflammatory process. Specifically, higher levels of IL-1RA, IL-8, IL-9, and IFN-γ were found at checkpoint 1 in patients with PMF compared to SS. The longitudinal analysis revealed a significant relationship between IL-1RA and MCP-1α and disease duration with MCP-1α also being associated with time and disease grade. Machine learning (ML) models were built using the cytokines selected through a sequential backward feature selection. The Support Vector Machine model achieved an accuracy of 83% in classifying disease stage (SS, PMF), and of 77% in predicting the disease progression. **Conclusions**: The findings suggest that cytokines can be used as dynamic biomarkers to reflect underlying inflammatory processes and monitor disease evolution. The performance of ML algorithms to predict diagnostic status based on cytokine profiles highlights their clinical value in supporting early diagnosis, monitoring disease progression, and guiding clinical decisions.

## 1. Introduction

Silicosis caused by exposure to crystalline silica dust from engineered stone is an emerging occupational lung disease that severely affects respiratory health [1]. Engineered stone (ES) is a material that is frequently used to manufacture kitchen and bathroom countertops, mainly composed of crystalline silica and synthetic resins, and its manipulation without the proper protections of health and safety has led to an occupational epidemic in some countries [2,3,4,5,6,7]. The inhalation of crystalline silica can produce silicosis, which can lead to progressive massive fibrosis (PMF) even years after the cessation of the exposure [8]. The latency period is shorter in engineered stone-related silicosis (ESS) compared to classic silicosis, and the disease presents itself with greater aggressiveness [9,10]. The main methods for diagnosing silicosis are chest X-rays (CXRs), high-resolution computed tomography (HRCT), clinical and occupational history, and pulmonary function tests (PFTs) [1,9].

Despite the techniques currently available in clinical practice, the early and accurate detection of silicosis remains a significant challenge [11]. Inter- and intra-observer variability in the interpretation of CXRs [12], the difficulty in interpreting radiological findings such as small opacities, in the early stages of the disease, limited access to medical imaging tests, high cost and the radiation exposure of HRCT [13,14] make it crucial to find new cost-effective tools that assist with diagnosis.

Very recently, biomarkers that could detect chronic inflammatory status are being explored by their potential to serve as a supportive tool for the diagnosis, monitoring, and early detection of silicosis progression [15,16,17,18,19,20,21,22]. In this context, cytokines have emerged as promising biomarkers, providing deeper insights into the mechanisms of disease and potentially improving both the diagnostic and prognostic accuracy. Cytokines are intercellular communication mediators that regulate inflammation, fibrosis, tissue repair, and a wide range of other cellular processes. The inflammasome-initiated cytokine cascade, triggered by the inability of macrophages and other immune cells in the alveoli to degrade silica particles due to their toxic nature, is responsible for the chronic inflammation and lung fibrosis characteristic of silicosis [23,24]. Our previous studies not only suggested that patients with ESS maintained a systemic inflammatory condition even years after cessation of exposure to silica dust [21] but also that IL-1RA, IL-8, IL-10, IL-16, IL-18, TNF-α, MIP-1α, G-CSF, and VEGF levels were increased in PMF patients compared to SS patients [25], positioning these cytokines as potential biomarkers of ESS severity.

Integrating artificial intelligence (AI) into clinical practice offers tools to improve disease diagnosis, reduce costs and time, and increase efficiency, leading to improvement in patient care [26,27]. Using AI to analyze blood test biomarkers has been shown to be highly effective in revealing hidden patterns and providing valuable insights [28,29,30]. Machine learning, a subfield of AI, has demonstrated the ability to capture complex multivariate relationships and interactions within healthcare data that may be difficult for humans to interpret [31]. Machine learning-based prediction models have already been developed for respiratory diseases such as COVID-19 and COPD as well as for other conditions such as diabetes, dementia, and breast cancer [32,33,34,35,36,37].

Although AI is increasingly being applied in healthcare and routine blood tests provide accessible biomarkers with diagnostic potential, the combined use of these approaches in patients with early-stage silicosis has only been minimally explored. This study aimed to analyze the association between cytokine profiles and disease severity and progression in patients diagnosed with ESS to use them as biomarkers of disease progression and to determine if they could contribute to the development of predictive tools to stratify risk and optimize clinical follow-up.

## 2. Materials and Methods

### 2.1. Subjects of the Study

A total of 72 subjects diagnosed with SS (*n* = 36) or with PMF (*n* = 36) were included in this study. Fourteen participants progressed from SS to PFM in a 5-year window relative to a control point. All participants were male workers with a history of cutting, polishing, and finishing ES countertops. They were part of a cohort of patients followed by the Department of Pneumonology of the Puerta del Mar University Hospital in Cádiz (Spain). Their diagnosis of ESS was performed based on their history of silica exposure, CXR, and/or HRCT, and, in some cases, by lung or mediastinal lymph node biopsy. Radiological findings were evaluated according to the ILO classification system [13] and the International Classification of HRCT for Occupational and Environmental Respiratory Diseases [38,39].

Patients were prospectively followed from 2017 to 2024. Demographic and clinical data were collected from the patient’s clinical history or by face-to-face interview during their medical appointment. Demographic information included the years marking the start and end of silica exposure, total duration of exposure (in years), and the year of diagnosis.

### 2.2. Ethics

This study was conducted following the Declaration of Helsinki and approved by the Research Ethics Committee of the Province of Cádiz, Spain (register numbers 151.22, 90.18, 157/16-SIL-2016-01, and 06.20). The Servicio de Salud Público de Andalucía (SSPA) Biobank of the Hospital Universitario Puerta del Mar (Cádiz, Spain) coordinated the collection, processing, and management of samples and clinical data according to the standard procedures established for this purpose. Informed consent was obtained from all subjects involved in the study.

### 2.3. Clinical Data

Four checkpoints were programmed for each patient in which pulmonary function was evaluated and blood samples were collected. The diagnosis status was also updated when necessary.

#### 2.3.1. Lung Function Measurements

Pulmonary function was assessed by certified technicians using standardized spirometry and diffusion equipment (Master Screen PFT/Body System, Jaeger-Viasys/CareFusion; or EasyOne Pro, ndd Medizintechnik AG). Measurements included forced vital capacity (FVC), forced expiratory volume in the first second (FEV_1_), the FEV_1_/FVC ratio, and the carbon monoxide diffusing capacity (DLCO). The latter was determined using the single-breath technique by established international guidelines.

#### 2.3.2. Plasma Cytokine Analysis

Vacutainer® EDTA tubes (Becton Dickinson, Madrid, Spain) were used to collect ten milliliters of venous blood samples. After two centrifugations, one at 1500× *g* for 10 min and the second at 2500× *g* for 15 min for platelet depletion, plasma fractions were obtained and stored at −80 °C until use. Twenty seven cytokines (FGF-basic, Eotaxin, G-CSF, GM-CSF, IFN-γ, IL-1β, IL-1RA, IL-2, IL-4, IL-5, IL-6, IL-7, IL-8, IL-9, IL-10, IL-12 (p70), IL-13, IL-15, IL-17A, IP-10, MCP-1, MIP-1α, MIP-1β, PDGF-BB, RANTES, TNF-α and VEGF) were analyzed following the instructions of the manufacturer using Bio-Plex Pro™ HumanCytokine 27-plex Assay (Bio-Rad Laboratories, Inc., Hercules, CA, USA) and Luminex technology FLEXMAP 3D^®^ equipment (Luminex Corporation, Austin, TX, USA). All samples were measured in duplicate.

### 2.4. Dataset

During the study, a total of 283 visits were recorded with four appointments per patient. The final dataset included 123 samples from patients with SS and 160 samples from patients with PMF due to 39% of SS patients progressing to PMF during the study period. The label associated with each sample (SS or PMF) for the development of the supervised machine models correspondeds to the patient diagnosis at the time of the blood test.

### 2.5. Statistical Methods

Cytokines presented concentrations below the lower limit of detection (LLOD) in more than 20% of the total number of samples. To mitigate the influence of extreme values, outlier correction was implemented using a one-sided winsorization technique. An upper outlier threshold was established at 3.5 standard deviations above the mean. Any value exceeding this threshold was replaced by the threshold value itself. This procedure preserved the full dataset while reducing the impact of high-value outliers on subsequent analyses.

To minimize the potential bias attributed to nondetectable—below the LLOD—or missing cytokine levels and pulmonary function values [40,41], imputation was performed by Multivariate Imputation by Chain Equations (MICE) [42].

Imputation using MICE offers a statistically robust way to estimate missing values by incorporating the relationships between variables in the dataset. This technique was chosen over simpler univariate methods (e.g., mean or median imputation) because it generates multiple complete datasets and uses predictive models to estimate missing values, thereby preserving the variance and correlation structure of the data, leading to more accurate statistical estimates. MICE works by creating a regression model for each variable with missing data, using all other variables as predictors. Regression models based on Bayesian Ridge regression were used. This approach’s Bayesian interpretation allows it to manage and quantify the uncertainty of the model’s coefficients. This feature makes it highly effective for imputing data in our study, where there are complex inter-variable correlations. We also enforced a constraint to ensure all imputed values were non-negative. The initial imputation for the missing values was performed using the mean. The algorithm successively estimated the missing values for each variable, subsequently employing the recently imputed variable to estimate the missing values for the subsequent variable, iterating this process until convergence was achieved. The entire process was repeated for 100 iterations to stabilize the imputations. In each iteration, previous imputations were updated with new predictions, improving their quality. A sensitivity analysis was undertaken to assess the robustness of the selected imputation technique. This analysis was conducted at checkpoint 1 and involved a comparison of the results obtained through imputation using MICE against four common imputation methods, specifically (a) complete case analysis; (b) simple imputation with 1% of the mean and median values of each cytokine; and (c) K-Nearest Neighbors.

Box plots were used to illustrate the distribution of marker levels across groups. To assess whether there were significant differences in cytokine expression levels and clinical variables between the patients with SS and PMF, a procedure combining normality testing, appropriate group comparisons, and multiple testing correction was followed. Before hypothesis testing, normality was assessed for each group using the Shapiro–Wilk test. If both groups met the normality assumption, an independent two-sample *t*-test was applied to evaluate mean differences. A non-parametric Mann–Whitney U test was used instead if the normality assumption was violated in either group. Pairwise correlations between selected cytokines and clinical variables were calculated using Spearman’s rank correlation coefficient, which does not require the assumption of a normal distribution.

Longitudinal changes in cytokine concentrations were analyzed using Generalized Linear Mixed-Effects Models (GLMMs), a well-suited approach for repeated-measures data, where *t*-tests cannot be used. The data were modeled with a Gamma distribution and a log-link function to account for the non-normal distribution of cytokine concentrations. The cytokine concentration was the dependent variable, while time (checkpoint), disease group (SS or PMF), and their interaction were included as fixed factors. To control for disease duration, the number of years since the first diagnosis was added as a covariate. Finally, to account for the non-independence of observations from the same individual, patient ID was included as a random effect (random intercept), which modeled subject-specific baseline variations. This methodology properly handled the inherent correlation in the data, ensuring the validity of the statistical analysis.

To account for multiple hypothesis testing and control the false discovery rate (FDR), *p*-values obtained from all tests were adjusted using the Benjamini–Hochberg procedure to control for the inflation of the type I error associated with multiple comparisons. The threshold for statistical significance was set at *p* < 0.05.

### 2.6. Machine Learning Models

The potential of artificial intelligence methods to classify cytokine samples from patients with SS or PMF was explored. Machine learning models were employed to identify combinations of cytokine levels that best predict a patient’s disease grade (discriminating between SS and PMF). Emphasis was placed on ensuring the generalizability and reliability of the predictive models, particularly given the longitudinal nature of the patient data.

Additionally, predictive models were developed to estimate each patient’s 5-year prognosis, aiming to project disease progression based on their cytokine profiles at various time points. This analysis was aimed at identifying biomarker patterns linked to both the current disease stage and future clinical outcomes. To achieve this, input features included not only the absolute cytokine levels at a specific time point but also their temporal variation.

Specifically, changes in cytokine concentration were calculated, comparing levels to the previous year and, when available, to two years prior. Additionally, the time from initial diagnosis at each checkpoint was included as a predictor. This strategy allowed the models to capture both the dynamic immune response patterns and the chronicity of the disease as they relate to future progression. This approach significantly enriched the feature space with temporal trends and clinical context, which has the potential to boost predictive performance.

A range of supervised machine learning classifiers were considered, including Decision Tree (DT), Random Forest (RF), Gaussian Naive Bayes (GNB), K-Nearest Neighbor (KNN), Linear Discriminant Analysis (LDA), Logistic Regression (LR) and Support Vector Machines (SVM) with a linear kernel.

#### 2.6.1. Decision Tree

A DT is a hierarchical supervised model represented as a tree where nodes are split into sub-nodes based on a threshold value of an attribute. Decisions are made at each node based on feature values, leading to the final prediction at the leaf nodes. The DT learns simple decision rules deduced from the training data. In this study, the DT model was built considering five as the minimum number of rows in a node and 10 as the maximum tree levels. Tree pruning was performed using 10-fold cross-validation to minimize errors. DTs have been used, for example, to identify lung cancer diagnosis using empirical negative control microRNAs [43] and to screen for serum biomarkers of silicosis [44].

#### 2.6.2. Random Forest

RF is built using many classification trees assembled based on the dataset. According to classification trees, tree votes are prepared for that class. The classified tree with major votes defines the forest structure. The RF algorithm also produces a tree, just like the DT, but several trees will be generated from the values of random samples in the dataset for this algorithm, and the final result will be based on the results of the majority of the trees developed. RF delivers significant improvements in the classification accuracy of a model by building a group of trees that generate results individually, collating those results, and picking the class that obtained the most votes [45].

#### 2.6.3. Gaussian Naïve Bayes

GNB is a statistical probabilistic machine learning algorithm that predicts class membership probabilities. GNB achieves high accuracy and speed when applied to a large dataset [46], but it also works very well on small datasets [47]. The Naïve Bayes algorithm has been used by several disease prediction models [34,35,36,37], and it achieves competitive accuracy in performing sentiment analysis [47,48].

#### 2.6.4. k-Nearest Neighbors

KNN is a nonparametric supervised learning algorithm used for classification that determines the class of new data by examining the majority class among the k-nearest neighbors. The input for the KNN algorithm involves the k-closest training instances in the training dataset. In recent years, KNN has been used in the diagnosis of silicosis with an electronic nose [49] and in the early prediction of lung tumors based on metabolomic biomarkers [43]. In this work, k values in the range from 2 to 100 were explored, using a four-fold CV strategy.

#### 2.6.5. Linear Discriminant Analysis

LDA is a classification technique that generalizes Fisher LD and finds the best linear combination of features to separate classes. LDA is a well-known machine learning algorithm that has been widely used in the respiratory medicine domain. It was recently used to predict overall survival and response to immunotherapy in non-small cell lung cancer [50] and to discriminate the breathprints of healthy subjects from COPD patients [51].

#### 2.6.6. Logistic Regression

LR is a widely used statistical model that allows multivariate analysis and the modeling of a binary dependent variable [52]. The multivariate analysis estimates coefficients like log odds or hazard ratios for each predictor included in the final model and adjusts them concerning the other predictors in the model. The coefficients quantify the contribution of each predictor to the output estimation [53]. LR models have been used to predict the development of hepatocellular carcinoma [54] and the recurrence of breast cancer after surgery [55].

#### 2.6.7. Support Vector Machines

SVM models are supervised machine learning methods that classify data samples by finding the appropriate hyperplane that best separates classes in a high-dimensional space [56]. SVM has recently been used in the respiratory field to predict early COVID-19 mortality with blood biomarkers [57] and to explore how immune cell infiltration contributes to chronic obstructive pulmonary disease (COPD) pathogenesis [58]. In this study, a linear kernel and a grid search for optimal parameters with a four-fold cross-validation (CV) were used for training the SVM classifier.

### 2.7. Data Preprocessing, Data Augmentation and Features Selection

Data standardization was applied for classifiers sensitive to feature scales (KNN, LDA, LR, and SVM).

A sequential backward feature selection technique (SBFS) was utilized to identify the most relevant set of characteristics for predicting disease progression and stage. This method enabled the identification of a set of predictors that optimized the performance of the classification model, avoided overfitting, and improved interpretability. At each step, the model performance was assessed through five-fold stratified cross-validation. This method ensured that all samples belonging to a single patient remained within the same fold for both training and testing, thus avoiding data leakage and providing a more robust estimate of the model’s ability to generalize.

Given that the dataset exhibited some class imbalance, SMOTE (Synthetic Minority Over-sampling Technique) was used in each fold to preserve the ratio of samples with SS and PMF [59]. Instead of simply duplicating existing samples, which could lead to overfitting, SMOTE creates new data points that are similar to the samples of the minority class already present in the data. SMOTE was applied exclusively to the training data within each cross-validation fold, ensuring that the model was not trained with information from the test set. This technique addressed potential class imbalance by generating synthetic samples only for the minority class, thus preventing the model from being biased toward the majority class.

The SBFS process started with the entire set of features. In each iteration, the performance of the model was evaluated for all possible subsets created by removing a single feature from the current set. For each subset and machine learning model, the average accuracy was calculated for the five cross-validation folds. The feature whose removal resulted in the highest average accuracy was deemed the least informative and was permanently removed. This process was repeated iteratively until only one feature remained, and the precision and identification of the features of each intermediate subset were recorded.

Upon completing the SFBS procedure, the subset that exhibited the highest overall accuracy throughout the process was selected as the optimal set of features for the final model. This approach is inherently model-dependent. The optimal subset of identified features is intrinsically related to the specific classifier used for evaluation. Since each classifier optimizes a distinct objective function, the set of features that yields optimal performance for a model may not necessarily be optimal for another. Consequently, this SBFS process generated a distinct subset of features for each classifier.

### 2.8. Performance Metrics and Validation

The metrics commonly used to assess the performance of machine learning models were calculated for all classifiers, including sensitivity (Se), specificity (Sp), area under the ROC curve (AUC), F1 score, precision (Pr), and accuracy (Acc). As mentioned above, five-fold stratified cross-validation was used to estimate the predictive performance of the models.

### 2.9. Software

Python 3.10 (Python Software Foundation, Wilmington, DE, USA) and SPSS 29 Statistics software (IBM Corp, Armonk, NY, USA) were used for statistical analysis and for training and validating the machine learning models.

## 3. Results

### 3.1. Characteristics of the Study Population

The sociodemographic and lung function data of the 72 patients with silicosis (SS and PMF) at the time of the first checkpoint are shown in Table 1, including age, duration of exposure, years since the beginning and end of exposure, years between exposure and diagnosis, and years of disease. All of these variables were similar with no significant differences between the SS and PMF groups. Four follow-up points were recorded during the study period, except for three patients with only three follow-ups and one with two. The mean age of the participants was 41.4 ± 7.1 years at baseline. Time since diagnosis at the first checkpoint of the follow-up averaged 5.7 ± 2.4 years.

A total of 283 visits were recorded during the study, averaging 3.93 visits per patient. This resulted in 283 unique data samples: 123 from patients diagnosed with SS and 160 from patients with PMF. All data samples were combined into a single dataset. Each sample was labeled for exploratory data analysis and model training using the categories SS and PMF.

Cytokines FGF-basic, GM-CSF, IL-1β, IL-2, IL-5, IL-6, IL-7, IL-10, IL-12 (p70), IL-15, IL-17, PDGF-BB, and VEGF were excluded from the analysis as they presented LLOD values in more than 20% of the samples. The quality of the resulting dataset was very high with the majority of variables demonstrating a notably low percentage of missing values (<10% per variable). The preliminary sensitivity analysis concerning imputation confirmed that MICE yielded highly stable results, as was expected given the low percentage of missing data and its capacity for preserving the underlying relationships in the data, leading to increased statistical power.

### 3.2. Lung Function Tests

Significant differences in mean values of FEV_1_/FVC, FEV_1_ (mL), FEV_1_ (%), and FVC (%) between the SS and PMF groups were observed at the first checkpoint.

All average values related to respiratory functions were lower in the group of patients diagnosed with PMF compared to those diagnosed with SS.

### 3.3. Cytokines

Table 2 presents the levels of inflammatory cytokines in peripheral blood plasma from patients diagnosed with SS and PMF at checkpoint 1, including adjusted *p*-values using the Benjamini–Hochberg correction.

**Figure 1:**
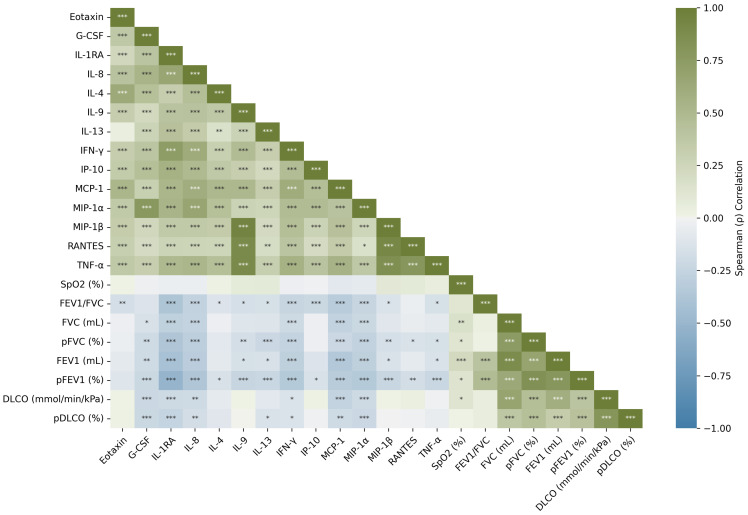
Spearman correlation heatmap between cytokines and clinical variables. Significance levels indicated using asterisks (* *p* < 0.05, ** *p* < 0.01, *** *p* < 0.001) based on the FDR-adjusted *p*-values. Abbreviations: G-CSF, granulocyte-colony stimulating factor; IL-1RA, interleukin-1 receptor antagonist; IL-4, interleukin 4; IL-8, interleukin 8; IL-9, interleukin 9; IL-13, interleukin 13; INF-γ, interferon-γ; IP-10, plasma interferon-γ-inducible protein 10; MCP-1, monocyte chemoattractant protein-1; MIP-1α, macrophage inflammatory protein-1α; MIP-1β, macrophage inflammatory protein-1β; RANTES, regulated upon activation normal T-cell expressed and secreted; TNF-α, tumor necrotic factor-α; SpO_2_, peripheral oxygen saturation; FEV_1_/FVC, proportion of forced expiratory volume in one second to forced vital capacity; FEV_1_, forced expiratory volume in the first second; FVC, forced vital capacity; DLCO, diffusing capacity of lung for carbon monoxide.

Spearman correlation analysis was performed to explore the monotonic associations between cytokine concentrations and clinical respiratory function parameters. The correlation matrix is presented in Figure 1 as a heatmap, where the strength and direction of the correlations are color-coded (green: positive, blue: negative), and statistically significant correlations are annotated with asterisks after FDR correction (*p* < 0.05). Several moderate-to-strong correlations were observed. Notably, G-CSF showed a strong positive correlation with MIP-1α (ρ = 0.77, *p* < 0.001); IL-1RA with IFN-γ (ρ = 0.74, *p* < 0.001); IL-9 with MIP-1β, RANTES, and TNFα (ρ = 0.95, 0.90 and 0.89 respectively, *p* < 0.001).

#### Cytokines Dynamics

**Figure 2:**
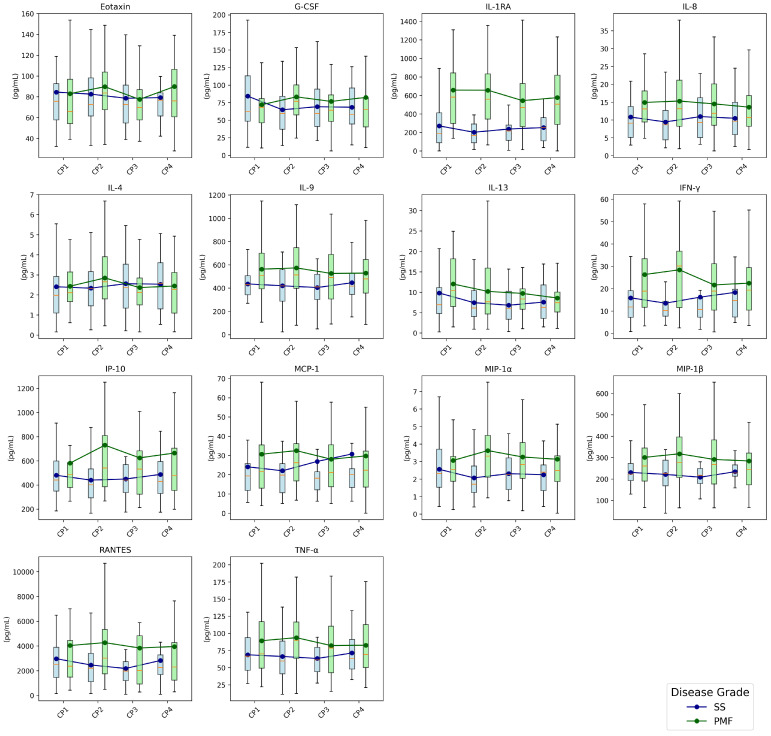
Longitudinal evolution of cytokine concentrations over the four checkpoints stratified by disease grade (SS and PMF). Box plots show the distribution of values and mean trends. The box plots show the distribution of the data, where the orange line represents the median, the box contains the data from the first to the third quartile, and the whiskers extend to show the full range of the data, excluding outliers. CPi: checkpoint *i*, i={1,2,3,4}. Abbreviations: SS, simple silicosis; PMF: pulmonary massive fibrosis; G-CSF, granulocyte-colony stimulating factor; IL-1RA, interleukin-1 receptor antagonist; IL-4, interleukin 4; IL-8, interleukin 8; IL-9, interleukin 9; IL-13, interleukin 13; INF-γ, interferon-γ; IP-10, plasma interferon-γ-inducible protein 10; MCP-1, monocyte chemoattractant protein-1; MIP-1α, macrophage inflammatory protein-1α; MIP-1β, macrophage inflammatory protein-1β; RANTES, regulated upon activation normal T-cell expressed and secreted; TNF-α, tumor necrotic factor-α.

To overview the temporal evolution of the inflammatory markers, box plots were generated for each cytokine at the four checkpoints, which were stratified by disease grade (Figure 2). Each box plot visualizes the distribution of cytokine levels at each time point with overlaid mean values connected by lines to highlight longitudinal trends. This provides a visual summary of all cytokine dynamics. Notably, patients with PMF exhibited higher levels of IL-1RA, IL-8, IL-9, IL-13, IP-10, IFN-γ, MIP-1α, MIP-1β, RANTES, and TNF-α along checkpoints compared to patients with SS.

GLMMs were used to analyze the longitudinal changes in the levels of each cytokine. The F-statistics, degrees of freedom, and *p*-values for cytokines detected as relevant for the fixed effects are provided in Table 3. The analysis revealed that the levels of IL-1RA and MCP-1α were significantly affected by at least one of the fixed factors. For IL1RA and MCP-1α, the number of years with the disease had a significant effect on cytokine levels. In particular, a significant interaction was found for MCP1α between time and disease grade.

As previously detailed, fourteen patients with SS progressed to PMF during the study period. Table 4 shows the mean levels of cytokines and pulmonary functions for this group of patients at the beginning of the study. No significant differences were found between the groups.

### 3.4. Machine Learning Models

Seven supervised machine learning models (DT, RF, GNB, KNN, LDA, LR, and SVM) were trained and validated to classify the disease stage (SS or PMF) and to estimate the 5-year prognosis of each patient.

#### 3.4.1. Disease Staging

To classify SS and PMF, models were trained and evaluated using cytokines, their longitudinal changes (denoted as $\Delta_{1}$ and $\Delta_{2}$ for changes between one and two checkpoints, respectively), and clinical information (years with the disease). SBFS was used to find the best subset of features in terms of accuracy. Evaluation was conducted using a stratified group 5-fold cross-validation strategy, incorporating SMOTE for handling class imbalance and appropriate feature scaling. Table 5 summarizes the classification performance and the selected best combinations of features for each model.

#### 3.4.2. Prognosis Prediction

Table 6 summarizes the performance obtained by machine learning models to predict the transition from SS to PMF in 5 years. The best combinations of cytokines estimated for each model are detailed. The performance of models was assessed using the same procedure as for disease staging models.

## 4. Discussion

It is well established that silicosis induces a chronic inflammatory state in the lungs. Inhaled crystalline silica particles are resistant to complete clearance by alveolar macrophages, triggering an ongoing inflammatory reaction that progressively leads to lung fibrosis. The disease continues to progress to more advanced stages even years after cessation of exposure [8,21,23,24]. Given this persistent inflammatory status, plasma/serum cytokines have been studied and reported as biomarkers in silicosis by several research groups [25,60,61,62,63,64,65,66,67,68,69]. Identifying which cytokines are altered in patients with ESS is key to understanding the physiopathology of this disease as well as guiding the development of future treatments.

A cohort of 72 patients with silicosis was followed from 2017 to 2024 for this study. Four visits per patient were recorded, resulting in a longitudinal follow-up of 27 cytokines related to the diagnosis for each patient. Thirteen cytokines were excluded from the analysis because they included more than 20% missing values.

A strong association of IL-1RA, IL-8, IL-9, and IFN-γ with the disease grade was found at checkpoint 1. Cytokine levels were higher in PMF patients than in SS patients (Table 2). These results are consistent with a state of persistent inflammation and pulmonary fibrosis characteristic of ESS. IL-1RA is an inhibitor of pro-inflammatory IL-1 and has been proposed as a good biomarker candidate for disease progression due to the increase in its levels as silicosis stages progress, which could be indicative of a mechanism to balance the effect of IL-1β [25,70]. Notably, some studies also suggest that the susceptibility and severity of silicosis could be related to some IL-1 polymorphisms, like IL-1RA +2018 [71,72]. IL-8 is a key pro-inflammatory mediator that acts as a potent neutrophil chemoattractant. It has been proposed as a good candidate marker to discriminate between disease stages, and its levels have been associated with progression and death in silicosis [60], with pulmonary impairment in copper smelter workers [73], and with progression in CWP disease [74]. IL-9 and IFN-γ are cytokines involved in the TH_1_ and TH_2_ responses with IL-9 promoting mastocytes and T cells growth and IFN-γ activating macrophages to promote pynocytosis and phagocytosis [75,76]. Furthermore, IL-9 is highly associated with respiratory diseases, and it has been suggested that the loss or alteration of the IFN-γ signaling pathway could be related to respiratory dysfunction and the development of fibrosis in silicosis [77]. These consistent and significant differences in cytokine levels between SS and PMF strongly suggest that these mediators are key components of the underlying physiopathology and are indicative of the extent of immune dysregulation. This observation is consistent with the understanding that the severity of the disease often reflects the intensity and nature of the inflammatory and immune responses.

The Spearman correlation heatmap showed a strong correlation between MIP-1β, RANTES, TNF-α and IL-9 (Figure 1). This result fits the previously expressed idea that TNF-α signaling could induce MIP-1β expression [78]. Furthermore, MIP-1β and RANTES are CC ligand chemokines linked to the CCR5 receptor, whose blockage has been proposed as a treatment for silicosis due to the inhibition of profibrotic chemokines [79]. Another pair with a significant correlation was G-CSF and MIP-1α, which are produced by macrophages and participate in neutrophil recruitment and growth [80,81]. Lastly, IL-1RA and IFN-γ also showed a significant correlation, having an indirect function linked to IL-1β levels, as a deficiency in IFN-γ would be associated with higher IL-1β and an elevated level of IL-1RA to balance its effect [77,82].

The longitudinal analysis revealed that IL-1RA and MCP-1α exhibited a significant relationship with the number of years after the diagnosis of the disease. In the case of MCP1α, we also found an association between time and disease grade (Table 3). MIP-1α is a chemokine that attracts and recruits macrophages, monocytes, and leukocytes to inflammatory sites. Elevated serum levels have been significantly correlated with pulmonary fibrosis and have been found in patients with systemic sclerosis, which is a disorder characterized by fibrosis and vascular changes [83]. IL-1RA acts by blocking the pro-inflammatory effects of IL-1, which may suggest that its increasing levels are part of a regulatory response to chronic inflammation associated with the disease.

During the study period, 39% of patients with SS progressed to PMF. This progression highlights the importance of identifying biomarkers that can predict disease evolution. However, no statistically significant differences at checkpoint 1 were found between the group of 22 patients who remained SS and the group of 14 patients who progressed from SS to PMF (Table 4).

Beyond individual cytokine analysis, we explored the utility of machine learning models to predict diagnostic status and prognosis based on cytokine profiles. Among all classifiers for disease staging, LDA demonstrated a strong overall performance, achieving an area under the ROC curve (AUC) of 0.847, an F1 score of 0.784, and an accuracy of 82.4%. This model incorporated a comprehensive feature set including IL-1RA, IL-8, IFN-γ, MCP-1, MIP-1β, RANTES, multiple first- and second-interval cytokine changes, as well as disease duration, indicating that both immune biomarker levels and their dynamic changes over time contribute significantly to disease classification. SVM also yielded high performance, with an AUC of 0.851, F1 score of 0.778, and accuracy of 82.7%. The SVM model selected a similar cytokine panel, with particular emphasis on IFN-γ, MIP-1β, and RANTES, and incorporated longitudinal changes and clinical duration as well. Similarly, LR and RF produced robust classification results, with AUCs of 0.839 and 0.812, and accuracies of 81.3% and 78.1%, respectively. Notably, the RF model achieved comparable performance using a compact feature set of only IL-1RA, IL-8, and RANTES. The KNN model, despite using a large and complex feature set spanning multiple cytokines and temporal deltas, showed solid performance (AUC = 0.807, accuracy = 77.5%) but with somewhat reduced specificity compared to LDA and SVM. On the other hand, GNB prioritized sensitivity (0.903), indicating a strong ability to identify PMF cases, although its specificity was lower (0.572), which may increase false positive rates. Finally, the DT classifier yielded the lowest performance among the models tested with an AUC of 0.729 and accuracy of 74.9%. Despite selecting a relatively broad panel of cytokines and their changes, DT appeared less capable of generalizing in this context compared to more sophisticated or ensemble approaches. Overall, these results highlight the importance of combining both static cytokine levels and their temporal changes, alongside clinical data, to improve the classification of SS versus PMF. The superior performance of LDA, SVM, and LR suggests that linear or margin-based classifiers effectively leverage these multidimensional data to distinguish disease severity. In addition, some of the cytokines selected for the disease staging models, such as IL-1RA, IL-8, TNF-γ, and MIP-1α, were also found to show differences between the groups of patients with SS and PMF in our previous study [25].

Concerning the machine learning models trained to predict the progression of five diseases from SS to PMF, the SVM classifier achieved the highest overall performance, with an AUC of 0.817, sensitivity of 0.740, specificity of 0.807, and accuracy of 77.2%, using a combination of baseline and $\Delta_{2}$ cytokine values along with the number of years with the disease. LR also performed well (AUC = 0.732, accuracy = 75.2%), using a more compact cytokine set. Although LDA attained a good accuracy of 70.4%, it required a more extensive array of cytokines. Interestingly, GNB showed the highest sensitivity (0.815) but at the cost of lower specificity (0.527) and accuracy (61.6%), suggesting that it may be useful for detecting positive cases but less effective overall. The RF model showed a remarkable specificity (0.916) but moderate precision (64.3%), while DT and KNN showed moderate results across metrics. These findings suggest that linear models (SVM and LR) are more robust for predicting the transition from SS to PMF when using both absolute cytokine levels and their longitudinal changes. The feature subsets selected by different models highlighted a consistent set of cytokines and their temporal changes that appear relevant for predicting disease progression. The presence of temporal change features ($\Delta_{1}$, $\Delta_{2}$) in nearly all models underscored that not only absolute cytokine levels but also their variation across disease checkpoints provide predictive information. Models such as DT and LDA selected a broader panel of cytokines and time-dependent features, potentially capturing more complex interactions but at the cost of interpretability. Conversely, models like KNN and LR selected more concise cytokine subsets emphasizing key markers such as G-CSF and RANTES. The GNB model uniquely incorporated MCP-1 and IP-10, suggesting that these cytokines may provide complementary predictive information. The number of years with the disease was selected as a relevant feature in both the LDA and SVM models, which demonstrated relatively high AUC values (0.746 and 0.817, respectively) compared to other models. This suggest that this clinical temporal variable contributes valuable information that enhances the models’ discriminative performance. By including disease duration, these models likely capture temporal progression dynamics that that may not be fully represented by cytokine levels alone. This is particularly relevant in the context of chronic diseases, where biomarker profiles can evolve over time. The superior performance of models including the time elapsed, in years, between the date of clinical diagnosis and the time of sample collection or clinical assessment, as reflected in metrics such as AUC and F1 score, indicates that this variable may improve patient stratification by accounting for risk or progression patterns associated with disease duration. Key immune mediators such as G-CSF, IL-9, IL-13, IFN-γ, and RANTES consistently contributed in models, confirming their biological relevance.

The selected cytokines in models for disease stage diagnosis highlighted IL-1RA, MIP-1β and RANTES as key biomarkers. Interestingly, RANTES and IL-1RA also stood out as markers for the prediction of prognosis, along with G-CSF and IL-13. These findings suggest that the complex interplay of multiple cytokines can provide a powerful signature to distinguish between diagnostic categories, suggesting that machine learning techniques could be valuable tools for clinical decision making, potentially aiding in the diagnosis, prognosis, and treatment strategies for silicosis. In general, models that incorporate both static and dynamic features tended to perform better, suggesting that monitoring cytokine trajectories over time is crucial for the early detection of disease evolution, and that the identified subsets of cytokines are candidate biomarkers for disease monitoring and stratification.

Finally, it is essential to acknowledge some limitations of this study. Our findings should be interpreted with the understanding that the model’s predictive performance is based on a single-center cohort with limited diversity and size. Furthermore, quantifying the environmental burden due to respirable silica dust throughout the duration of exposure in study participants was unattainable, but indirect data suggest that exposure to crystalline silica was very high in our cohort [84]. A multicenter longitudinal follow-up for this specific population is a crucial and necessary step for future research. This would not only enable the external validation of our model but would also facilitate the development and validation of new predictive tools in this under-researched area.

## 5. Conclusions

This study provides compelling evidence for a significant association between circulating cytokine levels and both the diagnostic status and progression of ESS. The identification of specific cytokines that vary over time, correlate with disease chronicity, and exhibit strong associations with disease grade offers valuable insights into the immunopathogenesis of ESS. These findings suggest that cytokines not only reflect the underlying inflammatory processes but may also serve as dynamic biomarkers of disease evolution.

Moreover, the successful application of machine learning algorithms to predict diagnostic status based on cytokine profiles underscores the potential clinical utility of these biomarkers in supporting early diagnosis, monitoring disease progression, and guiding clinical decision making. Such predictive modeling approaches may enhance the stratification of patients and enable more personalized disease management strategies.

Future research should prioritize the validation of these findings in larger and more diverse patient cohorts with longitudinal designs that allow the robust assessment of cytokine dynamics over time. Additionally, further investigation into the functional roles of key cytokines identified in this study could provide a deeper understanding of disease mechanisms and reveal novel therapeutic targets. Ultimately, the integration of cytokine profiling and computational modeling may contribute to the development of precision medicine approaches in the diagnosis and management of chronic occupational lung diseases such as ESS.

## Figures and Tables

**Table 1:** Sociodemographic data and pulmonary function values of patients with SS and PMF at the beginning of the study. Data are expressed as mean ± standard deviation. *p*-Values are presented after the Benjamini–Hochberg correction. *n* = number of cases (percentage). Significance levels indicated using asterisks (*p* < 0.05).

Data	SS (*n* = 36)	PMF (*n* = 36)	*p*-Value
Age	41.58 ± 7.75	41.86 ± 6.45	0.186
Years of exposure	13.75 ± 6.94	13.25 ± 6.22	0.157
Years since first exposure	21.06 ± 5.80	21.03 ± 5.80	0.171
Years since last exposure	7.25 ± 2.79	7.81 ± 2.83	0.086
Years from first exposure to diagnosis	15.75 ± 6.69	14.78 ± 5.81	0.143
Years from last exposure to diagnosis	1.86 ± 3.08	1.53 ± 3.51	0.200
Years since diagnosis	5.22 ± 2.51	6.17 ± 2.30	0.082
SpO_2_	97.4 ± 1.1	97.7 ± 1.0	0.441
FEV_1_/FVC	0.77 ± 0.06	0.73 ± 0.08	0.045 *
FEV_1_ (mL)	3388.06 ± 638.69	2958.88 ± 650.67	0.045 *
FEV_1_ (%)	90.10 ± 12.92	77.34 ± 16.24	0.005 *
FVC (mL)	4391.39 ± 736.80	4044.40 ± 824.71	0.156
FVC (%)	94.56 ± 12.34	85.52 ± 17.01	0.045 *
DLCO (mmol/min/kPa)	9.21 ± 1.97	8.54 ± 1.51	0.375
DLCO (%)	87.46 ± 17.90	80.41 ± 16.00	0.181

Abbreviations: SpO_2_, peripheral oxygen saturation; FEV_1_/FVC, proportion of forced expiratory volume in one second to forced vital capacity; FEV_1_, forced expiratory volume in the first second; FVC, forced vital capacity; DLCO, diffusing capacity of lung for carbon monoxide.

**Table 2:** Levels of inflammatory cytokines in plasma from peripheral blood in patients diagnosed with SS and PMF at checkpoint 1. *p*-values are presented after the Benjamini–Hochberg correction. Significance levels indicated using asterisks (* *p* < 0.05, *** *p* < 0.001).

Cytokine	SS (*n* = 36)	PMF (*n* = 36)	*p*-Value
Eotaxin	84.48 ± 41.81	83.09 ± 43.94	0.654
GCSF	84.17 ± 54.48	71.91 ± 43.26	0.474
IL-1RA	270.49 ± 244.48	657.37 ± 472.12	*p* < 0.001 ***
IL-4	2.40 ± 1.77	2.43 ± 1.22	0.474
IL-8	10.82 ± 7.50	14.92 ± 8.57	0.045 *
IL-9	435.70 ± 151.14	562.66 ± 261.17	0.045 *
IL-13	9.80 ± 7.53	12.00 ± 7.28	0.217
IFN-γ	15.90 ± 12.62	26.31 ± 20.20	0.045 *
IP-10	480.17 ± 184.34	580.93 ± 334.26	0.474
MCP-1	24.08 ± 22.35	30.64 ± 31.06	0.441
MIP-1α	2.56 ± 1.41	3.06 ± 2.03	0.441
MIP-1β	230.61 ± 65.29	301.07 ± 179.85	0.234
RANTES	2972.50 ± 1999.45	4040.01 ± 4389.79	0.942
TNF-α	68.77 ± 27.14	89.28 ± 53.67	0.376

Abbreviations: G-CSF, granulocyte-colony stimulating factor; IL-1RA, interleukin-1 receptor antagonist; IL-4, interleukin 4; IL-8, interleukin 8; IL-9, interleukin 9; IL-13, interleukin 13; INF-γ, interferon-γ; IP-10, plasma interferon-γ-inducible protein 10; MCP-1, monocyte chemoattractant protein-1; MIP-1α, macrophage inflammatory protein-1α; MIP-1β, macrophage inflammatory protein-1β; RANTES, regulated upon activation normal T-cell expressed and secreted; TNF-α, tumor necrotic factor-α.

**Table 3:** Longitudinal analysis of cytokine levels using generalized linear mixed models: fixed effects of time, disease grade, and disease duration in patients with SS and PMF. *p*-values are presented after the Benjamini–Hochberg correction. * denotes a statistically significant result after correction (*p* < 0.05).

Cytokine	Variable	F-Statistic	df	*p*-Value
IL-1RA	Checkpoint	2.9746	3	0.072
	Disease Grade	4.1158	1	0.072
	Years with Disease	7.6291	1	0.030 *
	Years with Disease × Disease Grade	1.6627	1	0.248
	Checkpoint x Disease Grade	0.9918	3	0.397
MIP-1α	Checkpoint	2.0350	3	0.171
	Disease Grade	2.2290	1	0.171
	Years with Disease	6.7780	1	0.040 *
	Years with Disease x Disease Grade	1.1840	1	0.277
	Checkpoint x Disease Grade	3.5050	3	0.040 *

Abbreviations: IL-1RA, interleukin-1 receptor antagonist; MIP-1α, macrophage inflammatory protein-1α; df, degrees of freedom; SS, simple silicosis; PMF, pulmonary massive fibrosis.

**Table 4:** Cytokine levels and pulmonary function values at checkpoint 1 of SS patients whose diagnosis did not change and of SS patients who ended up progressing to PMF over the next 5 years. Data are expressed as mean value ± standard deviation. *p*-values are presented after the Benjamini–Hochberg correction.

Data	SS (*n* = 22)	SS That Progress (*n* = 14)	*p*-Value
Eotaxin	75.10 ± 35.62	99.21 ± 47.69	0.661
G-CSF	69.96 ± 36.49	106.50 ± 70.44	0.661
IL-1RA	252.72 ± 211.39	298.42 ± 295.55	1.000
IL4	2.27 ± 1.76	2.61 ± 1.83	1.000
IL-8	9.98 ± 5.83	12.13 ± 9.67	1.000
IL-9	451.82 ± 175.83	410.37 ± 102.14	1.000
IL-13	10.69 ± 6.12	8.39 ± 9.42	0.661
INF-γ	15.69 ± 12.03	16.21 ± 13.96	1.000
IP-10	483.50 ± 192.86	474.94 ± 177.08	1.000
MCP-1	25.75 ± 26.97	21.46 ± 12.59	1.000
MIP-1α	2.30 ± 1.25	2.95 ± 1.60	1.000
MIP-1β	237.17 ± 72.20	220.31 ± 53.55	1.000
RANTES	3169.25 ± 2162.24	2663.34 ± 1744.25	1.000
TNF-α	69.28 ± 28.61	67.96 ± 25.68	1.000
SpO_2_ (%)	97.41 ± 1.05	97.39 ± 1.21	1.000
FEV_1_/FVC	0.78 ± 0.05	0.76 ± 0.07	1.000
FEV_1_ (mL)	3455.00 ± 631.81	3282.86 ± 658.73	1.000
FEV_1_ (%)	90.88 ± 12.48	88.88 ± 13.97	1.000
FVC_1_ (mL)	4397.73 ± 655.78	4381.43 ± 875.63	1.000
FVC_1_ (%)	94.44 ± 9.90	94.75± 15.87	1.000
DLCO (mmol/min/kPa)	9.23 ± 1.96	9.17 ± 2.05	1.000
DLCO (%)	86.39 ± 16.52	89.14 ± 20.42	1.000

Abbreviations: SS, simple silicosis; G-CSF, granulocyte-colony stimulating factor; IL-1RA, interleukin-1 receptor antagonist; IL-4, interleukin 4; IL-8, interleukin 8; IL-9, interleukin 9; IL-13, interleukin 13; INF-γ, interferon-γ; IP-10, plasma interferon-γ-inducible protein 10; MCP-1, monocyte chemoattractant protein-1; MIP-1α, macrophage inflammatory protein-1α; MIP-1β, macrophage inflammatory protein-1β; RANTES, regulated upon activation normal T-cell expressed and secreted; TNF-α, tumor necrotic factor-α; SpO_2_, peripheral oxygen saturation; FEV_1_/FVC, proportion of forced expiratory volume in one second to forced vital capacity; FEV_1_, forced expiratory volume in the first second; FVC, forced vital capacity; DLCO, diffusing capacity of lung for carbon monoxide.

**Table 5:** Performance metrics in the validation set for the machine learning models for disease stage classification.

Model	Selected Cytokines	Se	Sp	AUC	F1-Score	Pr	Acc
DT	IL-1RA, IL-13, IFN-γ, IP-10, MIP-1β, RANTES, $\Delta_{1}$IL-1RA, $\Delta_{1}$MIP-1α, $\Delta_{1}$RANTES, $\Delta_{2}$IL-4, $\Delta_{2}$IFN-γ, $\Delta_{2}$MIP-1β, $\Delta_{2}$RANTES	0.664	0.793	0.729	0.663	0.673	0.749
RF	IL-1RA, IL-8, RANTES	0.774	0.794	0.812	0.727	0.705	0.781
GNB	IL-1RA, IP-10, MIP-1β, $\Delta_{1}$MIP-1β, $\Delta_{1}$TNFα, $\Delta_{2}$IL-8	0.903	0.572	0.781	0.707	0.608	0.704
KNN	G-CSF, IL-1RA, IL-9, IL-13, IFN-γ, RANTES, TNFα, $\Delta_{1}$Eotaxin, $\Delta_{1}$G-CSF, $\Delta_{1}$IL-1RA, $\Delta_{1}$IL-8, $\Delta_{1}$IL-4, $\Delta_{1}$IP-10, $\Delta_{1}$MIP-1α, $\Delta_{1}$MIP-1β, $\Delta_{1}$RANTES, $\Delta_{2}$Eotaxin, $\Delta_{2}$IL-9, $\Delta_{2}$IL-13, $\Delta_{2}$IP-10, $\Delta_{2}$MIP-1α, $\Delta_{2}$MIP-1β, Years with the Disease	0.874	0.716	0.807	0.750	0.683	0.775
LDA	IL-1RA, IL-8, IFN-γ, MCP-1, MIP-1β, RANTES, $\Delta_{1}$Eotaxin, $\Delta_{1}$G-CSF, $\Delta_{1}$IL-1RA, $\Delta_{1}$IFN-γ, $\Delta_{1}$MCP-1, $\Delta_{1}$MIP-1α, $\Delta_{1}$MIP-1β, $\Delta_{1}$RANTES, $\Delta_{1}$TNF-α, $\Delta_{2}$IL-1RA, $\Delta_{2}$IL-4, $\Delta_{2}$IL-13, $\Delta_{2}$TNFα, Years with the Disease	0.879	0.778	0.847	0.784	0.725	0.824
LR	IL-1RA, IL-8, IL-4, IFN-γ, MCP-1, MIP-1β, $\Delta_{1}$Eotaxin, $\Delta_{1}$G-CSF, $\Delta_{1}$IL-9, $\Delta_{1}$TNF-α, $\Delta_{2}$IL-1RA, $\Delta_{2}$IL-4, $\Delta_{2}$IL-13, Years with the Disease	0.852	0.788	0.839	0.768	0.720	0.813
SVM	IL-1RA, IL-8, IFN-γ, IP-10, MCP-1, MIP-1α, MIP-1β, RANTES, $\Delta_{1}$IFN-γ, $\Delta_{1}$MIP-1β, $\Delta_{1}$RANTES, $\Delta_{2}$IL-1RA, $\Delta_{2}$IL-4, $\Delta_{2}$IL-13, Years with the Disease	0.840	0.821	0.851	0.778	0.741	0.827

Abbreviations: Se: sensibility; Sp: specificity; AUC, area under the ROC curve; Acc, Accuracy; DT, Decision Tree; RF, Random Forest; GNB, Gaussian Naive Bayes; KNN, K-Nearest Neighbors; LDA, Linear Discriminant Analysis; LR, Logistic Regression; SVM, Support Vector Machine; G-CSF, granulocyte-colony stimulating factor; IL-1RA, interleukin-1 receptor antagonist; IL-4, interleukin 4; IL-8, interleukin 8; IL-9, interleukin 9; IL-13, interleukin 13; INF-γ, interferon-γ; IP-10, plasma interferon-γ-inducible protein 10; MCP-1, monocyte chemoattractant protein-1; MIP-1α, macrophage inflammatory protein-1α; MIP-1β, macrophage inflammatory protein-1β; RANTES, regulated upon activation normal T-cell expressed and secreted; TNF-α, tumor necrotic factor-α.

**Table 6:** Performance metrics in the validation set for the machine learning models for prognosis prediction.

Model	Selected Cytokines	Se	Sp	AUC	F1-Score	Pr	Acc
DT	G-CSF, IL-8, IL-13, IFN-γ, RANTES, $\Delta_{1}$Eotaxin, $\Delta_{1}$G-CSF, $\Delta_{1}$IL-1RA, $\Delta_{1}$MIP-1α, $\Delta_{1}$TNF-α, $\Delta_{2}$Eotaxin, $\Delta_{2}$G-CSF, $\Delta_{2}$IL-8, $\Delta_{2}$IL-4, $\Delta_{2}$IL-13, $\Delta_{2}$IFN-γ, $\Delta_{2}$RANTES	0.562	0.836	0.699	0.549	0.544	0.765
RF	G-CSF, IL-9, IL-13, $\Delta_{1}$IL-1RA, $\Delta_{1}$IL-13, $\Delta_{1}$RANTES, $\Delta_{2}$G-CSF, $\Delta_{2}$IP-10, $\Delta_{2}$RANTES	0.535	0.916	0.674	0.572	0.643	0.800
GNB	IL-9, MCP-1, RANTES, $\Delta_{1}$IL-9, $\Delta_{1}$IL-13, $\Delta_{1}$MCP-1, $\Delta_{1}$RANTES, $\Delta_{2}$IL-1RA, $\Delta_{2}$IFN-γ, $\Delta_{2}$IP-10, $\Delta_{2}$RANTES	0.815	0.527	0.638	0.573	0.465	0.616
KNN	G-CSF, IL-9, $\Delta_{2}$RANTES	0.718	0.690	0.689	0.618	0.552	0.706
LDA	G-CSF, IL-1RA, IL-13, IFN-γ, IP-10, MIP-1α, RANTES, $\Delta_{1}$IL-1RA, $\Delta_{1}$IL-13, $\Delta_{1}$RANTES, $\Delta_{2}$G-CSF, $\Delta_{2}$IL-1RA, $\Delta_{2}$IL-8, $\Delta_{2}$IL-13, $\Delta_{2}$IFN-γ, $\Delta_{2}$MCP-1, $\Delta_{2}$MIP-1α, $\Delta_{2}$TNF-α, Years with the Disease	0.642	0.749	0.746	0.571	0.550	0.704
LR	G-CSF, IL-9, IL-13, IFN-γ, $\Delta_{1}$RANTES, $\Delta_{2}$G-CSF, $\Delta_{2}$RANTES	0.652	0.783	0.732	0.640	0.685	0.752
SVM	G-CSF, IL-1RA, IL-9, IL-13, IFN-γ, TNF-α, $\Delta_{2}$Eotaxin, $\Delta_{2}$IL-1RA, $\Delta_{2}$IL-4, $\Delta_{2}$IL-13, Years with the Disease	0.740	0.807	0.817	0.667	0.647	0.772

Abbreviations: Se: sensibility; Sp: specificity; AUC, area under the ROC curve; Acc, accuracy; DT, Decision Tree; RF, Random Forest; GNB, Gaussian Naive Bayes; KNN, K-Nearest Neighbors; LDA, Linear Discriminant Analysis; LR, Logistic Regression; SVM, Support Vector Machine; G-CSF, granulocyte-colony stimulating factor; IL-1RA, interleukin-1 receptor antagonist; IL-4, interleukin 4; IL-8, interleukin 8; IL-9, interleukin 9; IL-13, interleukin 13; INF-γ, interferon-γ; IP-10, plasma interferon-γ-inducible protein 10; MCP-1, monocyte chemoattractant protein-1; MIP-1α, macrophage inflammatory protein-1α; MIP-1β, macrophage inflammatory protein-1β; RANTES, regulated upon activation normal T-cell expressed and secreted; TNF-α, tumor necrotic factor-α.

## Data Availability

The data are not publicly available due to privacy or ethical restrictions. The data that support the findings of this study are available upon request from the corresponding author for researchers who meet the criteria for confidential data access, as stipulated by participant informed consent and the Institutional Research Ethics Committee of the province of Cadiz, Spain. Data requests can be made to this ethics committee via this email: ceic.hpm.sspa@juntadeandalucia.es.
